# Integrin α_D_β_2_ (CD11d/CD18) mediates experimental malaria-associated acute respiratory distress syndrome (MA-ARDS)

**DOI:** 10.1186/s12936-016-1447-7

**Published:** 2016-07-30

**Authors:** Isaclaudia G. de Azevedo-Quintanilha, Adriana Vieira-de-Abreu, André Costa Ferreira, Daniele O. Nascimento, Alessandra M. Siqueira, Robert A. Campbell, Tatiana P. Teixeira Ferreira, Tatiana M. Gutierrez, Gabriel M. Ribeiro, Patricia M. R. e Silva, Alysson R. Carvalho, Patricia T. Bozza, Guy A. Zimmerman, Hugo C. Castro-Faria-Neto

**Affiliations:** 1 Laboratório de Immunofarmacologia, Instituto Oswaldo Cruz, Fiocruz, Pavilhão Ozório de Almeida, Av. Brasil 4365, Manguinhos, Rio de Janeiro, RJ CEP 21045-900 Brazil; 2 Program in Molecular Medicine, Department of Internal Medicine, University of Utah, Salt Lake City, UT USA; 3 Laboratório de Inflamação, Instituto Oswaldo Cruz, Fiocruz, Pavilhão Ozório de Almeida, Rio de Janeiro, Brazil; 4 Laboratório de Engenharia Pulmonar no Programa de Engenharia Biomédica, Instituto Alberto Luiz Coimbra de Pós-Graduação e Pesquisa de Engenharia-COPPE/Universidade Federal do Rio de Janeiro, Rio de Janeiro, Brazil; 5 Laboratório de Fisiologia da Respiração, Instituto de Biofísica Carlos Chagas Filho, Universidade Federal do Rio de Janeiro, Rio de Janeiro, Brazil; 6 Programa de Produtividade Científica, Universidade Estácio de Sá, Rio de Janeiro, RJ Brazil

**Keywords:** Integrin α_D_β_2_, Acute lung injury, Acute respiratory distress syndrome, Inflammation, Malaria

## Abstract

**Background:**

Malaria-associated acute respiratory distress syndrome (MA-ARDS) is a potentially lethal complication of clinical malaria. Acute lung injury in MA-ARDS shares features with ARDS triggered by other causes, including alveolar inflammation and increased alveolar-capillary permeability, leading to leak of protein-rich pulmonary oedema fluid. Mechanisms and physiologic alterations in MA-ARDS can be examined in murine models of this syndrome. Integrin α_D_β_2_ is a member of the leukocyte, or β_2_ (CD18), sub-family of integrins, and emerging observations indicate that it has important activities in leukocyte adhesion, accumulation and signalling. The goal was to perform analysis of the lungs of mice wild type C57Bl/6 (a_D_^+/+^) and Knockout C57Bl/6 (a_D_^−/−^) with malaria-associated acute lung injury to better determine the relevancy of the murine models and investigate the mechanism of disease.

**Methods:**

C57BL/6 wild type (a_D_^+/+^) and deficient for CD11d sub-unit (a_D_^−/−^) mice were monitored after infection with 10^5^*Plasmodium berghei* ANKA. CD11d subunit expression RNA was measured by real-time polymerase chain reaction, vascular barrier integrity by Evans blue dye (EBD) exclusion and cytokines by ELISA. Protein and leukocytes were measured in bronchoalveolar lavage fluid (BALF) samples. Tissue cellularity was measured by the point-counting technique, F4/80 and VCAM-1 expression by immunohistochemistry. Respiratory function was analysed by non-invasive BUXCO and mechanical ventilation.

**Results:**

Alveolar inflammation, vascular and interstitial accumulation of monocytes and macrophages, and disrupted alveolar-capillary barrier function with exudation of protein-rich pulmonary oedema fluid were present in *P. berghei*-infected wild type mice and were improved in α_D_β_2_-deficient animals. Key pro-inflammatory cytokines were also decreased in lung tissue from α_D_^−/−^ mice, providing a mechanistic explanation for reduced alveolar-capillary inflammation and leak.

**Conclusions:**

The results indicate that α_D_β_2_ is an important inflammatory effector molecule in *P. berghei*-induced MA-ARDS, and that leukocyte integrins regulate critical inflammatory and pathophysiologic events in this model of complicated malaria. Genetic deletion of integrin subunit α_D_ in mice, leading to deficiency of integrin α_D_β_2_, alters lung inflammation and acute lung injury in a mouse model of MA-ARDS caused by *P. berghei*.

## Background

Malaria is an infectious disease that is caused by the genus *Plasmodium* sp. transmitted through the bite of Anopheles mosquitoes that are infected with protozoan parasites and is a major public health problem 40 % or more of the global population is at risk for malaria [1]. The pathogenesis of malaria is multi-factorial, with both host and *Plasmodium* sp. factors playing critical roles [2, 3]. Nevertheless, the mechanisms responsible for the high morbidity and mortality of severe cases of malaria remain poorly understood. In endemic areas, many infections in semi-immune population present as an uncomplicated febrile illness. In more severe cases, non-immune individuals may exhibit a number of syndromes including severe anaemia (SA), cerebral malaria (CM) or respiratory distress syndrome [4, 5].

Lung involvement in malaria has been described most often in non-immune individuals, with infection by *Plasmodium falciparum* [6–9], *Plasmodium ovale* [7, 10, 11], *Plasmodium vivax* and *Plasmodium malariae* [6, 7, 12–16]. While the alveoli and airways can also be involved in mild infection [17], acute alveolar injury and acute respiratory distress syndrome (ARDS) are major sequelae of severe malaria and have significant morbidity and mortality [3, 17–19]. Malaria-associated ARDS (MA-ARDS) has been reported in infection with all human malarial parasites, although the greatest number of cases is caused by *P. falciparum* and *P. vivax* [17, 20, 21]. Patients with alveolar involvement in malaria have classically been reported to have pulmonary oedema, with recent recognition that the alveolar oedema is due to increased pulmonary capillary permeability [21–24]. In addition to altered alveolar-capillary barrier function with leak of protein-rich oedema fluid [3, 22–24], which is a cardinal manifestation of acute alveolar inflammation, alveolar involvement in human malaria has other significant inflammatory components, including leukocyte accumulation [17, 21, 25–29]. Alveolar inflammation is also a fundamental feature of ARDS induced by bacterial sepsis, infectious pneumonia, aspiration of gastric contents, major trauma, and other common ‘triggers’ [30]. A difference is that, in these more common etiologies of ARDS, alveolar injury is thought to be primarily caused by neutrophil- and platelet-dependent damage to endothelial and epithelial barriers of the alveolar-capillary membrane [30, 31], whereas in MA-ARDS monocytes and macrophages dominate in the inflammatory infiltrate [21, 25–29]. MA-ARDS has been modelled in studies utilizing mice and other animals, yielding mechanistic insights and experimental correlates [21, 32–37]. There is evidence that lung injury in the murine model *Plasmodium berghei* ANKA strain [33, 34, 37, 38] is associated with intravascular sequestration of parasitized red blood cells [33, 39], suggesting that it is a useful surrogate for human malarial disease [26, 40].

Integrins are cell surface heterodimers formed by non-covalent association of α and β polypeptide chains (sub-units). Integrins are widely expressed on mammalian cells and have multiple activities in cellular adhesion, migration, signalling, and fate [41, 42]. A sub-family of integrins, termed the β_2_, CD18, or leukocyte integrins, share a common β_2_ sub-unit and are expressed on circulating and tissue leukocytes [43, 44]. Four α chains pair with the β_2_ peptide sub-unit to yield four leukocyte-restricted integrins: α_L_β_2_ (CD11a/CD18, LFA-1), α_M_β_2_ (CD11b/CD18, MAC-1, CR3), α_X_β_2_ (CD11c/CD18), and α_D_β_2_ (CD11d/CD18) [43, 44]. Leukocyte β_2_ integrins are required for host defence against many pathogens and for tissue surveillance and repair, as demonstrated by deficiency syndromes that cause recurrent infections and impaired wound healing in humans and animals [44, 45]. In contrast, however, β_2_ integrin-mediated activities of leukocytes also contribute to tissue injury in a variety of inflammatory syndromes [46].

Integrin α_D_β_2_, the most recently identified β_2_ integrin, is expressed on human and murine leukocytes, although its basal expression is different in man and mouse [47–51]. Integrin α_D_β_2_ is expressed on tissue leukocytes in human inflammatory syndromes, including atherosclerosis [48], arthritis [52], and ARDS [51]. In rodents, there is evidence that α_D_β_2_ can be induced on macrophages or monocytes in the spleen and liver [50], lung [53], and blood [54] in response to inflammatory challenge, and that α_D_β_2_ contributes to inflammatory tissue damage in experimental spinal cord and brain injury [55–57]. Previously, Miyazaki et al. [50] found that genetic deletion of α_D_ in mice, leading to deficiency of α_D_β_2_, alters survival and systemic cytokine levels in mice infected with *P. berghei* without altering parasitaemia or anaemia. α_D_β_2_ influences the pathogenesis of experimental cerebral malaria in *P. berghei* infection (unpublished studies). In this work was examined α_D_β_2_ in lung involvement in *P. berghei* -infected animals and found that it influences key features of acute lung injury in this model of experimental MA-ARDS.

## Methods

### Mouse models of malaria

The Animal Welfare Committee of the Oswaldo Cruz Institute approved the experiments in these studies under the licence number L-033/09. Wild Type (a_D_^+/+^) and C57BL/6 deficient for CD11d integrin (a_D_^−/−^) mice [50] weighing 20–25 g were obtained from the Oswaldo Cruz Foundation breeding unit and used throughout the study. The animals were kept at constant temperature (25 °C) with free access to food and water in a room with a 12-h light/dark cycle. C57BL/6 mice were infected by an ip injection of 200 mL of PBS containing 10^5^ red blood cell (RBC) parasitized with the Pasteur strain of *P. berghei* ANKA [58]. All analyses were performed at day 7 post-infection. Between 90 and 100 % of infected mice developed lung injury and cerebral malaria (data about cerebral malaria not yet published).

### Bronchoalveolar lavage fluid analysis

Infected and uninfected mice were euthanized using isoflurane (Abott Labs do Brasil LTDA) and bronchoalveolar lavage fluid (BALF) from both lungs was performed by instillation and aspiration of 1 mL of cold 1× phosphate buffered saline (PBS) [33]. Total leukocytes (diluted in Turk’s 2 % acetic acid fluid) were counted using Neubauer chamber hemocytometer. Differential counts were performed in cytospins (Cytospin3, Shandon, CA, USA) and stained by the May-Grünwald-Giemsa method. The BALF was spun at 350 g at room temperature for 5 min, and the supernatant was removed and stored at −80 °C for further analyses. BALF total protein concentration was measured using a BCA protein assay kit (Thermo Scientific, Waltham, MA, USA).

### Lung permeability

Mice were injected intravenously (iv) with 0.2 mL of 2 % Evans Blue dye (Sigma-Aldrich Brasil LTDA, São Paulo, Brazil) and were sacrificed 1 h later. Before collecting lung, mice were perfused with PBS 1X. The lungs were collected and placed in 2 mL of formamide (VETEC Química Fina LTDA, Duque de Caxias, RJ, Brazil) at 37 °C, overnight to extract Evans Blue dye from the tissue [59]. Absorbance was measured at λ = 620 nm (Molecular Devices Spectra Max 190, Sunnyvalle, CA, USA). Evans Blue dye concentration was calculated from a standard curve and is expressed as mg of Evans Blue dye per lung tissue.

### Lung histology

Before collecting lung, mice were perfused with PBS 1X. Lungs from infected and uninfected mice were inflated by injecting 1.0 mL of 10 % buffered formalin through the same catheter used to perform BALF. Lungs were removed fixed in formalin and embedded in paraffin. Lungs sections of 5-mm thickness were stained with haematoxylin-eosin [59]. Analysis of tissue sections was performed in a Olympus BX41 microscope (Melville, NY, USA) at a magnification of ×200. The number of mononuclear cells in lung tissue was determined by the point-counting technique across 20 random, non-coincidents microscopic fields in an Olympus BX41 microscope at a magnification of ×1000 [60].

### Cytokine determinations

Before collecting lung, mice were perfused with PBS 1X. Lungs of infected and uninfected mice were excised and homogenized in 750 mL of a protease inhibitor cocktail (Complete, mini EDTA-free Roche Applied Science, Mannheim, Germany) for 30 s, using a Ultra-Turrax Disperser T-10 basic (IKA-Guangzhou, China). Homogenates were stored at −20 °C, for analysis of cytokines using a commercial ELISA kit according to the manufacturer’s instructions (R&D Systems Duo set kits, Minneapolis, USA).

### Quantitative RT-PCR

Before collecting lung and spleen, mice were perfused with PBS 1X. Extraction of total RNA from lungs and spleen was performed using TRIzol^®^ (Invitrogen, Carlsbad, CA, USA), according to the manufacturer’s instructions. After extraction, RNA concentration and quality were determined using a NanoDrop 2000 spectrophotometer (Thermo Scientific). One microgram of total RNA was reverse-transcribed to single-strand cDNA using the SuperScript First-Stand (Invitrogen). a_D_ transcripts in the cDNA pool obtained from the reverse transcriptase reaction were quantified by real-time quantitative fluorogenic PCR. TaqMan Universal PCR Master Mix (Applied Biosystems, Foster City, CA, USA) was used to quantify gene expression according to the manufacturer’s instructions. RNA expression levels were calculated using the Data Assist Software v.3, and normalized against the expression levels of the housekeeping gene hypoxanthine guanine phosphoribosyl transferase (HPRT) [50]. The primers used were as follows: a_D_ (TaqMan-murine-Mm01159115_m1) and HPRT (TaqMan-murine- Mm01545399_m1).

### Immunofluorescence

The primary monoclonal antibodies used in immunofluorescence reactions were VCAM-I (rat anti-Mouse CD106, eBioscience, San Diego, CA, USA) and negative control (rat anti-IgG2b, Bioscience). Before collecting lung, mice were perfused with PBS 1X. Lung tissues from infected and uninfected mice a_D_^+/+^ and a_D_^−/−^ were frozen in tissue freezing medium (TBS™, Triangle Biomedical Science, Inc, Durham, NC, USA) and 10-mm cryostat sections were deposited on 3-aminopropyl triethoxysilane (Sigma-Aldrich) prepared slides. The sections were then fixed in 2 % paraformaldehyde and permeabilized with 0.01 % Triton X-100 (Sigma) for 10 min. Sections were then blocked with 10 % normal goat serum, for 40 min and subsequently incubated with primary antibody overnight at 4 °C. Alexa 546-conjugated goat anti-rat antibody was applied 1/1000 dilution and incubated for 40 min. The sections were then layered with anti-fade medium conjugated with DAPI (Vectashield, Vector Laboratories, Burlingame, CA, USA). The slides were analysed by confocal laser scanning microscopy on Zeiss LSM 510-META (Jena, Germany).

### Immunohistochemistry

Primary monoclonal antibodies used in immunohistochemistry reactions were F4/80 (MCA497-Serote c; anti-rat) and IgG-HRP (STAR72-Serotec; goat anti rat) were also used in immunohistochemistry reactions. After deparaffinization, the sections were hydrated with TBS (Tris/HCl 0.05 M + NaCl 0.5 M, pH 7.6), and H_2_O_2_ in methanol 3 % was add for 15 min. Slides were washed with TBS and blocked with Tris–HCl + BSA 5 % for 2 h and subsequently incubated with primary antibody diluted Tris–HCl + BSA 1 % for 12 h at 4 °C. After incubation, the slides were washed with TBS. The secondary antibody HRP-conjugated was diluted in Tris–HCl and incubated for 2 h. The revealed was made with 3-amino-9-ethylcarbazole (AEC) for 15 min. The slides were washed with distilled water, counterstained with haematoxylin of Mayer and mounted in an aqueous medium-containing gelatin. The slides were analysed using a Olympus BX41 microscope at a magnification of ×200.

### Airway hyperreactivity analysis

Airway hyperreactivity (AHR) [61] was analysed in infected and uninfected mice using non-invasive whole-body plethysmography (Buxco, Sharon, Connecticut, CT, USA) 7 days’ post infection. AHR was measured after aerosolization of 1× PBS followed by increasing concentrations of methacholine (0, 171, 6, 25 mg/mL; Sigma-Aldrich) for 7 min into the chamber. AHR was expressed as an average enhanced pause [38]. There was an interval of 10 min between each aerosol exposure and within this period of time the Penh values had returned to baseline.

### Lung pressure/volume analysis in mechanically ventilated mice

Pressure/volume (PV) relationships was examined in mechanically ventilated wild type and α_D_^−/−^ uninfected and PbA-infected in mice. After 40 s of stabilization period under baseline settings (V_T_ of 8–10 mL/kg, RR of 80 breaths/min, ratio I:E of 1:1 and PEEP of 2 cm H_2_O), RR was decreased to 6 breaths/min, PEEP of 0 cmH_2_O, I:E ratio set at 4:1, V_T_ was increased to 25–30 mL/kg and three PV curves were obtained. Airway pressure was continuously measured with a differential pressure transducer (UT-PDP-50, SCIREQ, Canada) at the distal end of the endotracheal tube (ETT). Airflow was measured with a heated-controlled pneumotachograph connected to a pressure transducer and positioned between the ETT and the Y-piece of the mechanical ventilator. Paw and airflow signals were low-pass filtered at 30 Hz, digitized at 1000 Hz and recorded with a purpose-built software (Data Acquisition System) written in LabVIEW^®^ (National Instruments, Austin, TX, USA). Volume was then calculated by numerical integration of airflow. The PV curves where peak airway pressure remains stable and near to 20 cm H_2_O were fitted with a sigmoidal model (Eq. 1):

1 $${\text{V }} = {\text{ a }} + \, \left[ {{{\text{b}} \mathord{\left/ {\vphantom {{\text{b}} {\left( { 1 { } + {\text{ e}}^{{( - {\text{ Pel}}\left( {\text{V}} \right) - {\text{c}}/{\text{d}})}} } \right)}}} \right. \kern-0pt} {\left( { 1 { } + {\text{ e}}^{{( - {\text{ Pel}}\left( {\text{V}} \right) - {\text{c}}/{\text{d}})}} } \right)}}} \right]$$

where Pel is the elastic pressure, V is the Volume, and a, b, c and d are the coefficients of the model, obtained by nonlinear fitting with the Levenberg–Marquardt method. The point of maximal compliance (PMC) is equivalent to the parameter c of this model and was estimated for each animal.

### Statistical analysis

Statistical analysis was carried out using the GraphPad Prism software (San Diego, CA, USA). P values were calculated by unpaired Student’s t test, except for PMC calculated with Wilcoxon rank sum test. Results are expressed as mean ± SEM (median (IQR)). The level of significance was set at P ≤ 0.05.

## Results

### Expression of *α*_*D*_ mRNA transcripts is increased in the lung in *Plasmodium berghei* infection

**Fig. 1:**
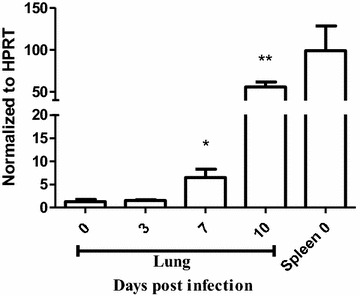
α_D_ mRNA expression is increased in the lungs of mice infected with *Plasmodium berghei* ANKA. Infected wild type were sacrificed 7 or 10 days later. Lungs were removed, processed and reverse transcribed. Transcripts for *α*_*D*_ were quantified by real-time quantitative PCR and normalized to the levels of hypoxanthine guanine phosphoribosyl transferase (*HRPT*). Expression of *α*_*D*_ in spleen was used as a positive control. Each *bar* represents the mean ± SEM of determinations in tissue from 5 animals. *P ≤ 0.04, **P ≤ 0.001 by student’s t test

Transcripts for *α*_*D*_ in the lungs of wild type mice were examined in the basal state and after infection with *P. berghei*. RT-PCR analysis of mRNA extracted from lungs of naïve, uninfected animals demonstrated a low level of the *α*_*D*_ transcript. The expression of *α*_*D*_ increased dramatically at 7 and 10 days after infection with *P. berghei* and approached levels detected in the spleen (Fig. 1), where α_D_β_2_ integrin is constitutively expressed [50].

### Disrupted alveolar-capillary membrane barrier function and alveolar inflammation are ameliorated in α_D_β_2_-deficient mice

**Fig. 2:**
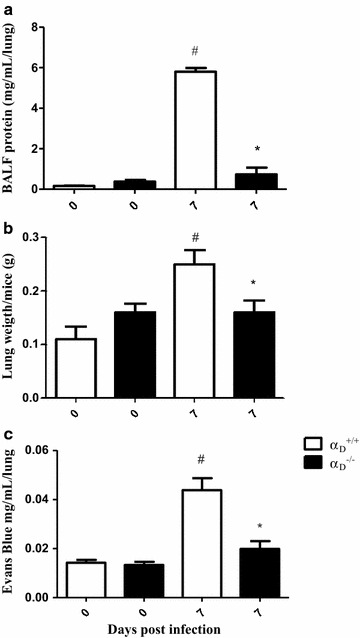
Alveolar-capillary barrier disruption and increased permeability pulmonary are ameliorated in α_D_β_2_-deficient mice infected with *Plasmodium berghei* ANKA. Infected wild type (α_D_^+/+^) and α_D_β_2_-deficient (α_D_^−/−^) were study 7 days after infection. Bronchoalveolar lavage fluid (BALF) was performed. **a** Total protein concentration was measured in BALF samples using a BCA protein determination assay. **b** A second group of animals was sacrificed without BALF and the lungs were removed and weighed immediately after sacrifice. **c** In a third group of α_D_^+/+^ and α_D_^−/−^ mice, Evans Blue Dye (2 %, 0.2 mL) was injected intravenously. The animals were sacrificed 1 h later, and Evans Blue Dye concentration in the lung tissue. Each *bar* in panels **a**–**c** indicates the mean ± SEM. of determinations from 5 animals.^#^P ≤ 0.05 compared to the respective control group;  P ≤ 0.05 compared to α_D_^+/+^ infected mice

**Fig. 3:**
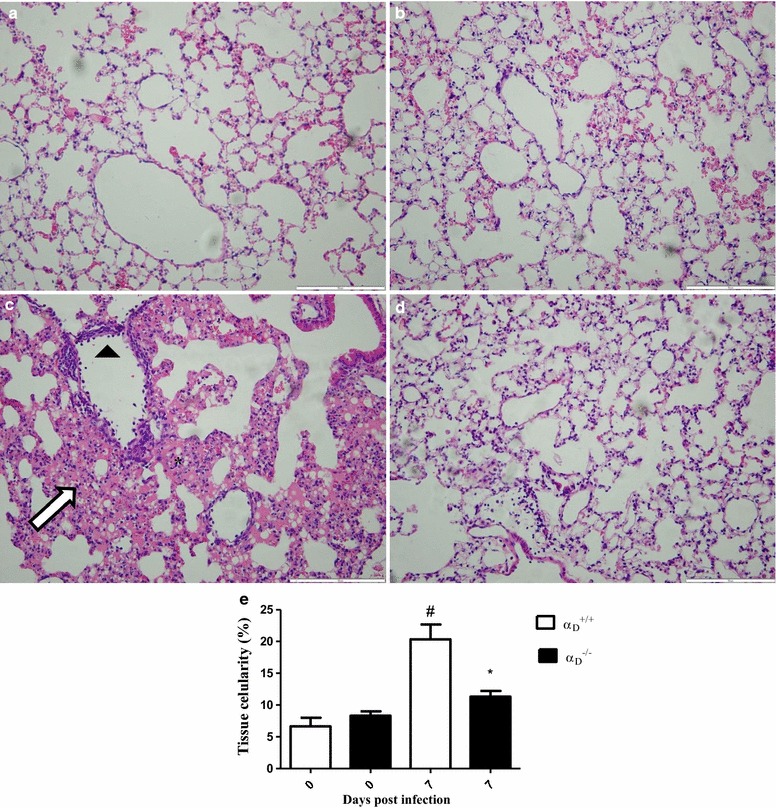
Vascular and interstitial inflammation are key components of MA-ARDS in *Plasmodium berghei* ANKA-infected mice. After staining with haematoxylin-eosin, the sections were examined by optical microscopy (×200 magnification). The *scale bars* indicate 50 µm. **a** Lung tissue from an uninfected α_D_^+/+^ mouse. **b** Lung section from an uninfected α_D_^−/−^ animal. **c** Lung tissue from an infected wild type α_D_^+/+^ animal. The *arrowhead* identifies adherent leukocytes in a pulmonary vessel, indicating vascular inflammation. Diffuse interstitial infiltrates (*arrow*) and focal haemorrhages (*asterisks*) were also seen. **d** Lung tissue section from an infected α_D_^−/−^ mouse. The features illustrated in **a**–**d** are representative of those seen in lung tissue from 3 individual mice in each condition. **e** Lung cellularity was determined by the point counting technique across 20 random, non-coincident microscopic fields at magnification of ×1000, as described in “Methods” section. Each *bar* represents the mean of determinations in tissue from 3 animals ± SEM.^#^P ≤ 0.05 compared to respective control

Infection with *P. berghei*, induces alveolar-capillary barrier disruption and lung edema in mice of several backgrounds [33–37]. Consistent with these observations protein concentration in BALF samples (Fig. 2a), lung weight (Fig. 2b), and lung endothelial permeability as measured by extravasation of Evans Blue dye (Fig. 2c) were increased in wild type mice infected with *P. berghei*. Similar alterations in these variables are seen in murine acute lung injury induced by other insults [62] and are key correlates of disrupted alveolar-capillary barrier integrity and increased permeability pulmonary oedema, which are cardinal features of clinical and experimental ARDS [30, 31, 62]. Each variable was improved to near basal levels in α_D_^−/−^ mice infected with *P. berghei* compared to measurements in infected wild type animals (Fig. 2a–c), indicating that alveolar-capillary barrier disruption is ameliorated in mice deficient in α_D_β_2_. Focal haemorrhages, which were scattered throughout the inflamed lung parenchyma of P*. berghei*-infected wild type mice (Fig. 3), were less frequent in α_D_β_2_-deficient mice. The latter finding is also consistent with amelioration of endothelial leakiness in α_D_^−/−^ animals.

**Fig. 4:**
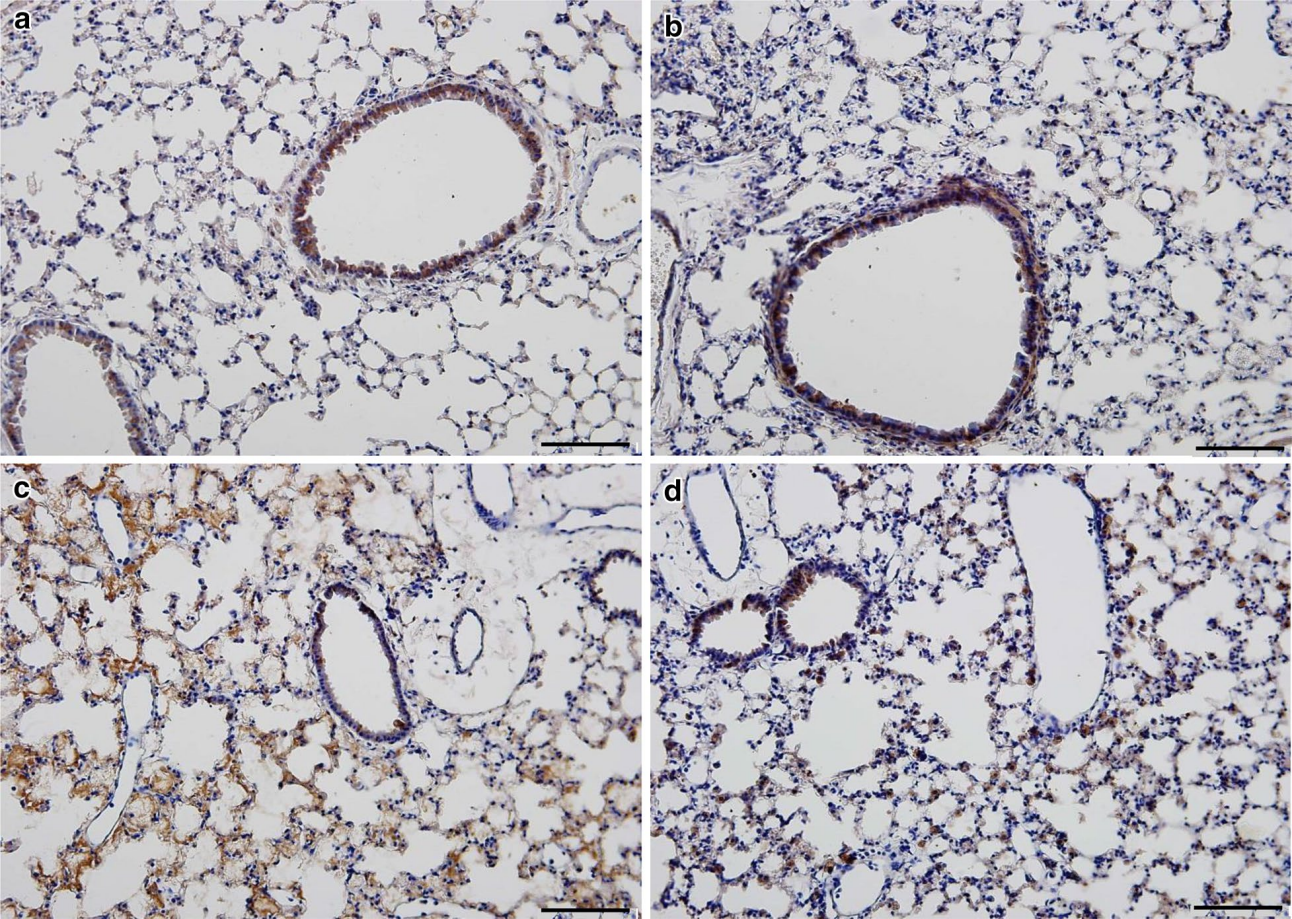
F4/80-positive leukocytes accumulate in the alveoli in *Plasmodium berghei* ANKA-induced experimental MA-ARDS and are reduced in α_D_β_2_-deficient mice. Lung tissue from α_D_^+/+^ and α_D_^−/−^ mice infected with *P. berghei* was harvested at 7 days after infection. Lung slices were incubated with anti-F4/80 as the primary antibody and HRP-conjugated secondary antibody. The sections were examined and photographed using an Olympus BX41 microscope at ×200 magnification (*scale bars* 200 mm). **a** Sections from a control α_D_^+/+^ mouse. **b** Section from a control α_D_^−/−^ mouse. **c** Lung tissue from an infected α_D_^+/+^ wild type animal. **d** Section from an infected α_D_^−/−^ mouse. The staining patterns shown in **a**–**d** are representative of those in lung tissue from 3 individual mice for each condition

Accumulation of leukocytes was found in the lungs of wild type mice infected with *P. berghei* (Fig. 3). Nevertheless, increased leukocyte numbers was not detected in the alveolar spaces as demonstrated by cell counts in BALF samples (total BALF cell numbers in wild type *P. berghei*-infected mice 4.86 × 10^5^ ± 0.7502 cells/mL compared to 4.165 × 10^5^ ± 0.4847 cells/mL in uninfected wild type controls P = 0.4863). This finding, and microscopic analysis (Figs. 3, 4), indicated that the primary accumulation of leukocytes is in the alveolar vessels and interstitium rather than in the alveolar space under these conditions. This distribution of inflammatory cells in the lungs was also reported in previous studies of *P. berghei*-infected C57BL/6 mice [33]. To better characterize leukocytes that accumulated in the alveolar interstitium of mice infected with *P. berghei,* lung sections from infected animals were stained with mAb F4/80 and high frequency of positive cells was observed (Fig. 4). This indicates that macrophages and monocytes are the major leukocyte types accumulating in the lungs of *P. berghei*-infected animals at 7 days post-infection [63, 64]. In conclusion, the findings in this model are similar to descriptions of alveolar inflammation and accumulation of monocytes and macrophages in alveolar vessels and the interstitium in histopathologic specimens from the lungs of patients who died with MA-ARDS [3, 21, 25, 28, 29].

Lung cellularity and alveolar leukocyte accumulation were dramatically reduced in samples from α_D_^−/−^ mice infected with *P. berghei* compared to leukocyte accumulation in lungs of infected wild type animals (Fig. 3). Consistent with this, the frequency of F4/80-positive leukocytes was reduced in α_D_β_2_-deficient mice (Fig. 4). In addition, leukocytes adherent to the endothelium of pulmonary vessels were reduced in lung sections from infected α_D_^−/−^ mice when compared to sections from infected wild type animals (Fig. 3).

**Fig. 5:**
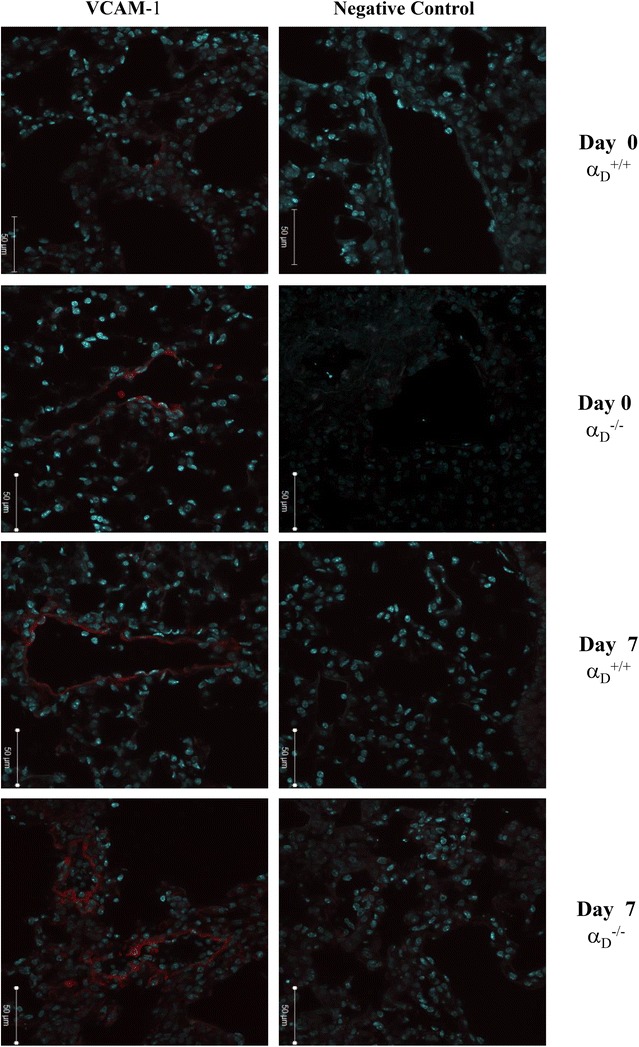
VCAM-1 expression is increased in the lungs of wild type and α_D_β_2_-deficient mice after infection. Wild type and α_D_β_2_-deficient mice were infected with PbA and lungs were harvested at sacrifice at 7 days of infection. Lungs from infected wild type animals at day 0 were also obtained and handled in the same fashion. Staining for VCAM-1 was accomplished with a specific fluorescently labelled rat anti-mouse antibody (red staining). A rat anti-mouse IgG2b was used as an irrelevant control antibody. Nuclei were stained with DAPI. The sections were examined and photographed by immunoflourescent microscopy at ×20 magnification (50 μm). The patterns shown in the panels are representative of those seen in samples from 3 individual animals for each condition

To further characterize the decreased cellularity and leukocyte accumulation in the lungs of α_D_^−/−^ mice, expression of VCAM-1, an important ligand for α_D_β_2_ [50, 65, 66] was examined. Increased expression of VCAM-1 was observed in the lungs of infected animals. Increased VCAM-1 staining was similar in the α_D_^+/+^ and α_D_^−/−^ genotypes (Fig. 5), indicating that expression of this adhesive ligand is not altered by the genetic manipulation and suggesting that the decrease in leukocyte accumulation in the lungs of α_D_^−/−^ animals (Figs. 3, 4) is due to deficiency of α_D_β_2_.

### Inflammatory cytokines are decreased in the lungs of α_D_β_2_-deficient mice infected with *Plasmodium berghei*

**Fig. 6:**
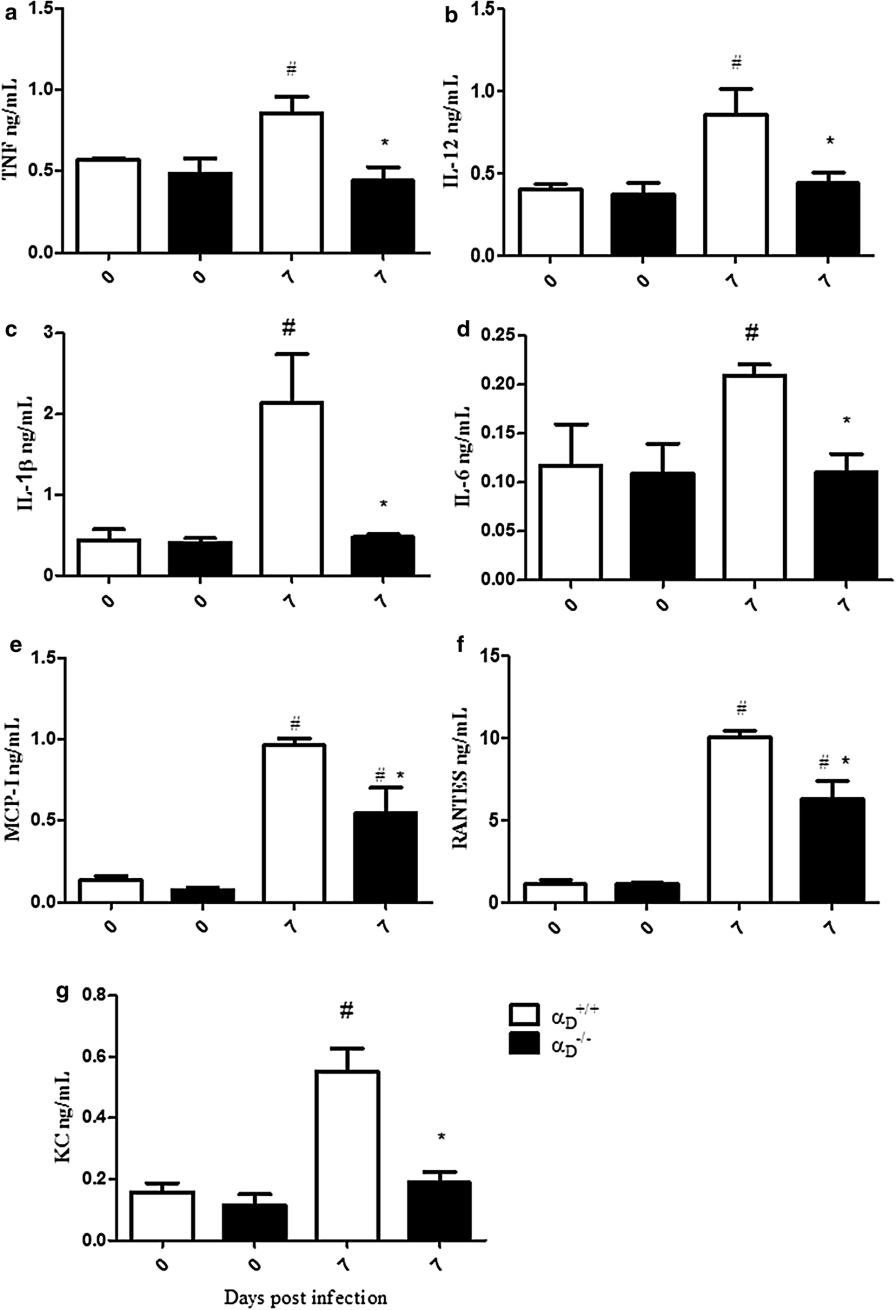
Inflammatory cytokines are decreased in the lungs of α_D_β_2_-deficient mice in *Plasmodium berghei* ANKA-induced MA-ARDS. Wild type and α_D_β_2_-deficient mice were infected with PbA and lungs were harvested at 7 days after infection. Cytokine and chemokine levels were measured in the homogenates by ELISA. **a** TNF. **b** IL-12. **c** IL-1b. **d** IL-6. **e** MCP-I. **f** RANTES. **g** KC. Each *bar* indicates the mean ± SEM. of determinations in lung samples from 5 individual animals.^#^P ≤ 0.05 compared to the respective control group; *P ≤ 0.05 compared to infected wild type (α_D_^+/+^) mice

To explore mechanisms related to decreased acute lung injury and inflammation in α_D_β_2_-deficient mice with experimental MA-ARDS, inflammatory cytokines were measured in lung homogenates from α_D_^−/−^ and wild type animals collected 7 days after *P. berghei* infection. Concentrations of tumour necrosis factor (TNF), interleukin-1β (IL-1β), interleukin 6 (IL-6), interleukin-12 (IL-12), monocyte chemotactic protein 1 (MCP-1), regulated upon activation normal T cell expressed and secreted (RANTES), and the murine orthologue of interleukin-8 (IL-8), KC, were increased in lungs of wild type α_D_^+/+^ animals (Fig. 6). The level of each of these cytokines was lower in lung lysates from α_D_β_2_-deficient mice, and in some cases was similar to that detected in samples from uninfected control animals (Fig. 6).

### Airway hyper-responsiveness and obstruction are induced by *Plasmodium berghei* infection and are improved in *P. berghei*-infected mice deficient in α_D_β_2_

**Fig. 7:**
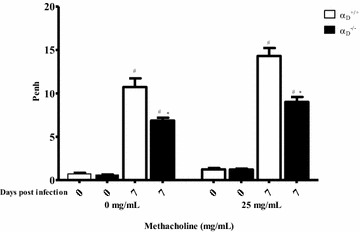
*Plasmodium berghei* ANKA infection induces increased airway reactivity that is ameliorated in α_D_β_2_-deficient mice. Infected α_D_β_2_-deficient and wild type mice were studied 7 days after infection. Uninfected α_D_^−/−^ and α_D_^+/+^ animals were also studied as controls. Airway hyperreactivity was evaluated by challenge of the animals with aerosolized phosphate buffered saline (PBS) followed by methacholine (25 mg/mL in PBS) and is expressed as average enhanced pause (Penh). Each *bar* represents determinations in 10 animals (mean ± SEM).^#^P ≤ 0.05 compared to the respective control group; *P ≤ 0.05 compared to infected α_D_^+/+^ mice

Although alveolar-capillary membrane injury and MA-ARDS are the principal features of pulmonary involvement in malaria [21] some patients have evidence for airway dysfunction, including cough and airway obstruction documented by spirometry and pulmonary function assessment [6, 17]. *P. berghei*-infected mice showed increased airway reactivity that is further increased by methacholine challenge at 7 days post-infection (Fig. 7). The physiologic alterations in the basal state and the enhanced responses to methacholine indicate airway hyper-responsiveness [67] in wild type α_D_^+/+^ animals infected with *P. berghei*. In infected α_D_^−/−^ mice airway hyper-responsiveness was abrogated in both the basal state and after methacholine administration (Fig. 7).

### Lung pressure/volume relationships are altered by *Plasmodium berghei* infection

**Fig. 8:**
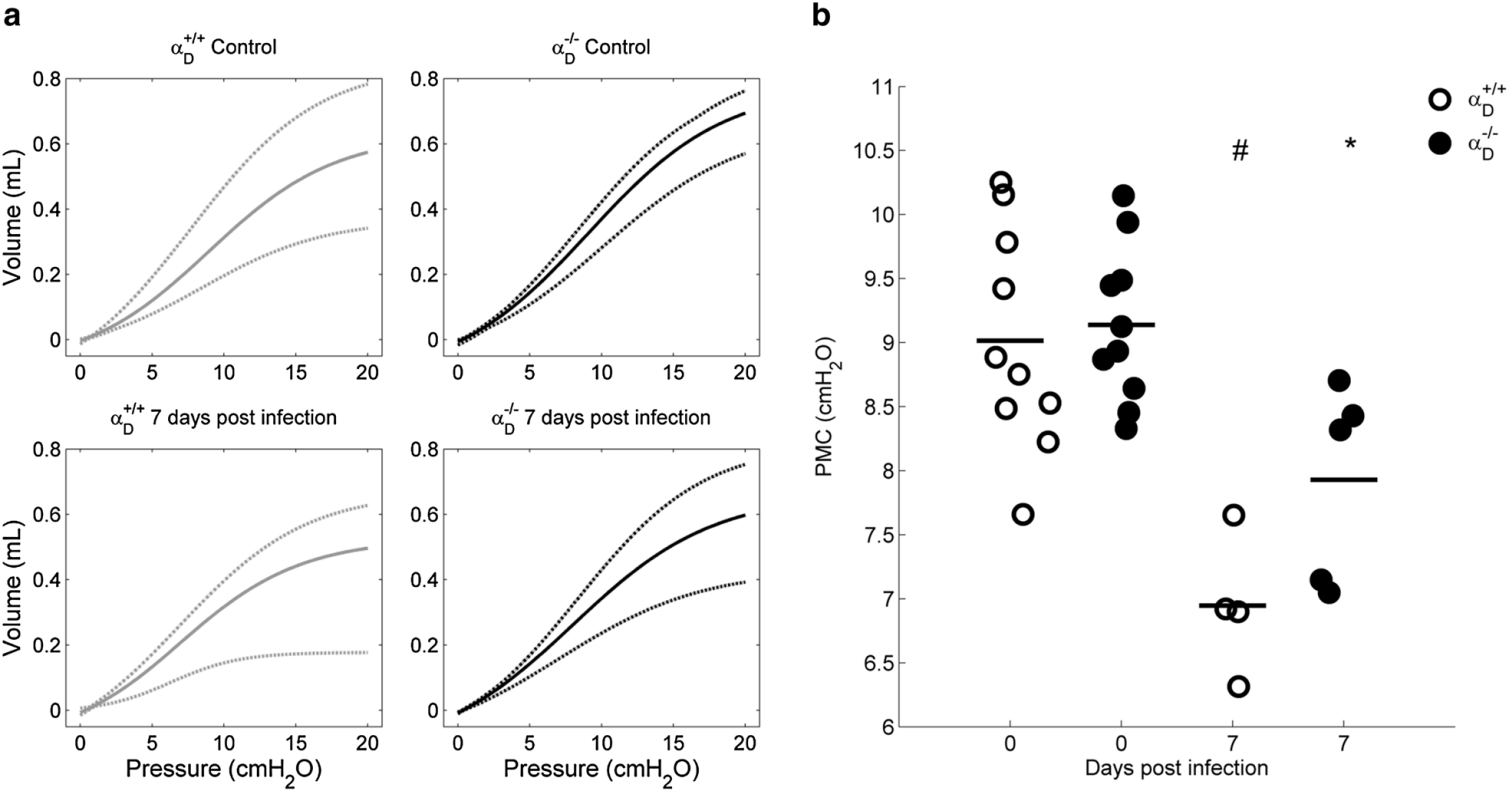
Pressure/volume curves are sigmoidal and PMC is decreased in *Plasmodium berghei* ANKA-infected mice. Wild type and α_D_^−/−^animals from infected and control groups were mechanically ventilated and three PV curves were obtained (RR = 6 breaths/min, PEEP = 0 cm H_2_O, I:E ratio = 4:1, V_T_ = 25–30 mL/kg). The PV curves where peak airway pressure remains stable and near to 20 cm H_2_O were fitted according to Eq. 1. The curve model gives the pressure value at which the respiratory system has maximum compliance. **a** *Upper* (*dashdot*), mean (*solid*) and *lower* (*dashed*) fitted curves of each group. **b** The PMC are lower, for infected wild type (P < 0.002) and α_D_^−/−^ (P < 0.008) mice compared with respective uninfected controls

All PV curves were sigmoidal and PMC values were lower in infected groups. The expected sigmoidal shape of **t**he PV curves indicated a predominance of cyclic recruitment at the lower portion of the curve followed by a linear region and a predominance of hyperdistention in the upper portion (Fig. 8a). In infected mice of both genotypes, respiratory system compliance was decreased with lower volumes at equal pressures, with an increase in hyperdistention and a decrement of the PCM pressure (Fig. 8b). There was a trend toward improvement (P = 0.064) in the PMC values for infected α_D_^−/−^ mice (Fig. 8b).

## Discussion

Complicated malaria is a major challenge in management of malarial infections, which are dominant global public health problems [3]. Pulmonary complications are among the most serious and potentially lethal consequences of malaria [18, 19, 22], and it is clear that they occur in human malarial infections caused by parasite species in addition to *P. falciparum*, including *P. vivax* and *Plasmodium knowlesi* [17, 20, 21, 26, 28, 29]. MA-ARDS is the most fulminant syndrome of lung involvement in malaria [18, 19, 21] and, like ARDS induced by other infectious and non-infectious causes [30, 31, 68], is characterized by generation of pro-inflammatory cytokines, acute alveolar inflammation, disrupted alveolar capillary membrane barrier function, and increased permeability pulmonary oedema [19, 21]. Alveolar involvement in MA-ARDS may be due in part to organ-specific, local intravascular inflammation and unique events such as release of toxins from parasitized RBCs sequestered in the lung [37, 69, 70]. Mouse models have the potential to reveal key mechanistic features and common and divergent organ-specific responses in MA-ARDS, cerebral malaria, and other complicated malarial syndromes [21, 40, 69]. The present study provides additional evidence that acute alveolar injury in the *Plasmodium berghei* ANKA model of MA-ARDS has features similar to those in humans with clinical MA-ARDS [19, 21], including increased permeability pulmonary oedema, vascular and interstitial inflammation with accumulation of alveolar monocytes and macrophages, focal parenchymal haemorrhages, and pulmonary generation of cytokines. In addition, AHR and obstruction are elements of lung involvement in the *P. berghei* model, as they are in some patients with complicated and uncomplicated clinical malaria [6, 17, 19]. Moreover, *P. berghei* infection alters respiratory system elastic components, consistent with alveolar and airway inflammation. It is important to note that changes in PV relationships are late responses to progressive pulmonary involvement, and with a more extended time course this tends to worsen. Together, these measurements indicate that *P. berghei* infection induces substantial alterations in physiologic lung and airway function that accompanies lung inflammation and oedema. Finally, this model was used to examine regulation of key events in *P. berghei*-induced lung inflammation by an important leukocyte integrin, α_D_β_2_.

β_2_ integrins have critical activities in leukocyte biology, including regulation of adhesion, targeting and accumulation in infected or injured tissue sites, apoptosis, activation and inflammatory signalling, and immune interactions [42, 44, 45, 71]. Thus, β_2_ integrins are members of a complex fabric of effector molecules that regulate leukocyte participation in infectious, inflammatory and immune host responses. Integrin α_D_β_2_ is the most recently identified β_2_ integrin [44, 48], and its specific contributions to infectious and inflammatory pathologies are largely unexplored. Experiments in this study indicate that it has major effector activities in experimental MA-ARDS induced by *P. berghei*. Expression of transcripts coding for the α_D_ subunit were increased in the lungs of mice infected with *P. berghei*. Increased *α*_*D*_ transcripts may have been due to accumulation of *α*_*D*_-expressing monocytes or other *α*_*D*_-expressing leukocytes from the blood [54], induction of *α*_*D*_ expression in resident alveolar macrophages and other lung myeloid leukocytes [50, 53], or both mechanisms. While this issue remains to be resolved, increased expression of *α*_*D*_ in the lungs of animals with experimental MA-ARDS implies upregulation of α_D_β_2_ and that α_D_β_2_ on lung leukocytes has important activities in this condition. Of interest, α_D_β_2_ is increased on the surfaces of leukocytes in the lungs of human subjects who died of ARDS triggered by other causes [51].

This study used mice with genetic deletion of *α*_*D*_ and consequent absence of α_D_β_2_ [50] to examine its contributions to the pathophysiology of *P. berghei*-induced MA-ARDS. Specific blocking antibodies against the α_D_ subunit or α_D_β_2_ are not commercially or generally available, making a genetic approach essential to target α_D_β_2_ while leaving other leukocyte integrins intact [42, 44]. This study shows that key determinants of acute lung injury were improved in α_D_^−/−^ animals at 7 days after infection. The seven-day time point was chosen because, in previous study of severe malaria caused by *P. berghei*, survival curves for wild type and α_D_^−/−^ mice began to diverge at 7 days [50]. In the current experiments, lung leukocyte accumulation was reduced in α_D_^−/−^ at 7 days. This finding is consistent with published evidence that α_D_β_2_ mediates leukocyte accumulation and monocyte migration in tissue inflammation and injury in vivo [55, 57, 72], and that it mediates macrophage adhesion to tissue ligands [50] and purified protein targets [73] in vitro. In parallel, pulmonary vascular leak, evaluated by Evans Blue Dye extravasation [21, 62, 74], and increased permeability alveolar oedema, assayed by BALF protein concentration and lung weight [21, 62], were ameliorated in α_D_β_2_-deficient animals. Thus, key determinants of the pathology and pathophysiology of acute alveolar injury in experimental and clinical ARDS [30, 62, 68] were improved in α_D_^−/−^ animals in comparison to assessment of these variables in wild type mice. Furthermore, there was a similar pattern of improvement in airway function in α_D_β_2_-deficient compared to wild type mice. AHR has not previously been examined in rodent models of malaria-induced pulmonary involvement. While airway hyperreactivity is not a prominent feature of ARDS triggered by sepsis and other common causes of ARDS [31, 68], cough, other symptoms of airway obstruction, and physiologic evidence for small airway narrowing and reactivity have been detected in patients with malaria [6, 7, 17, 19]. These findings suggest that airway dysfunction may contribute to the pathophysiology of MA-ARDS. Improvement in airway function in α_D_^−/−^ mice indicates that airway inflammation is a mechanism of airway dysfunction in the *P. berghei* model. A trend in improvement was found in PV relationships in α_D_^−/−^ mice, although this was not statistically significant.

Chemokine and cytokine levels were also reduced in the lungs of infected α_D_^−/−^ mice compared to the levels in pulmonary tissue from wild type mice with MA-ARDS. This may be a key mechanism in improved alveolar inflammation, reduced alveolar-capillary leak, and reduction in alveolar oedema in α_D_β_2_-deficient animals. Pro-inflammatory cytokines, including IL-1β, TNF, IL-8/KC, and others, are synthesized by alveolar macrophages and monocytes and are thought to drive alveolar inflammation and injury in experimental and clinical ARDS [30, 31, 68]. Cytokine imbalance is proposed to be a feature of the pathophysiology of clinical MA-ARDS [19]. In the studies reported here, intrapulmonary IL-1β, TNF, and KC were substantially reduced in infected α_D_^−/−^ mice. Similarly, IL-12 and RANTES, which have pleiotropic activities in inflammation, were also reduced, as was MCP-1, which recruits monocytes to the lungs [75, 76]. Thus, reduced pro-inflammatory cytokines in infected α_D_^−/−^ mice may in part account for reductions in the vascular and interstitial inflammation in these animals. Reductions in key cytokines may also account for improvement in alveolar-capillary barrier integrity and reduced leak of protein and fluid into the alveolar spaces of α_D_β_2_-deficient mice compared to wild type mice with MA-ARDS. IL-1β and TNF are major agonists for endothelial VE cadherin internalization and endothelial barrier disruption in models of inflammatory injury, including experimental ARDS [77–79]. TNF and IL-1β were reduced to baseline levels in the lungs of infected α_D_^−/−^ mice, potentially contributing to improved barrier function. Of note, a reduced systemic cytokine levels was also found in α_D_β_2_-deficient mice infected with *P. berghei* in earlier analysis of this model [50]. The molecular events that mediate altered chemokine and cytokine levels in α_D_β_2_-deficient mice are not yet completely defined. Nevertheless, in studies with human monocytes were found that engagement of α_D_β_2_ with activating antibodies or specific ligands induces outside-in signaling to chemokine and cytokine synthetic pathways [51]. These evolving observations indicate that α_D_β_2_, like other β_2_ integrins [80, 81], is a key regulator of chemokine and cytokine gene expression in myeloid leukocytes.

Conversely, the release of cytokines, leads to cell activation and increased expression of adhesion molecules such as integrins and immunoglobulins superfamily members [82–84]. Several studies show the role of integrins in development pulmonary oedema, including beta integrins [85]. The main functions of CD11/CD18 integrins are adhesion and migration [86, 87], and previous studies showed that VCAM-1 is an important adhesion receptor in models of experimental [88] and human malaria [89–91] and is a potential ligand for leukocytes and PRBC, in cytoadherence processes that lead to obstruction of the microcirculation. This work demonstrate that the absence of CD11d integrin does not interfere in VCAM-1 expression, and suggest that the effects described in this work are due to an increased expression of CD11d integrin and not by a lack of its ligand. This study with VCAM-1 suggests increased endothelial activation during *P. berghei* infection, however, more experiments are necessary to better characterize this activation.

## Conclusion

In conclusion, this report demonstrate that integrin α_D_β_2_ is an important effector molecular in the inflammatory manifestations of severe *P. berghei* infection, including increased alveolar-capillary membrane permeability, alveolar monocyte and macrophage accumulation, and lung oedema, and that it is a key determinant of critical pathophysiologic events in this model of MA-ARDS. Blunting of acute lung injury in α_D_β_2_-deficient mice likely contributes to the early survival advantage of α_D_^−/−^ animals in lethal *P. berghei* infection, although they ultimately succumb to progressive hyperparasitaemia and severe malarial anaemia [50]. Other specific integrin heterodimers on a variety of cell types [41–43] may also contribute to the pathobiology of malarial infection, although this has been examined in only a limited fashion [92, 93]. Because of their diverse activities in inflammatory cell adhesion, accumulation, and signalling, α_D_β_2_ and other leukocyte integrins may have pivotal roles in MA-ARDS, cerebral malaria and other complications of experimental and clinical malarial infection [44, 45, 71]. Dissection of the roles of leukocyte integrins will likely provide new insights into the innate and adaptive immune fabric of the host responses to Plasmodium, and increased understanding of cell- and organ-specific events in malarial inflammation.

## Abbreviations

MA-ARDS: malaria-associated acute respiratory distress syndrome
ARDS: acute respiratory distress syndrome
RBC: red blood cell
BALF: bronchoalveolar lavage fluid
AHR: airway hyperreactivity
PV: lung pressure/volume
ETT: endotracheal tube
PMC: point of maximal compliance
PBS: phosphate buffer saline
HPRT: hypoxanthine guanine phosphoribosyl transferase
